# The Relationship Between Caffeine Intake and Dry Eye Disease

**DOI:** 10.1097/ICO.0000000000002979

**Published:** 2022-01-26

**Authors:** Morten Schjerven Magno, Tor P. Utheim, Mathias Kaurstad Morthen, Harold Snieder, Nomdo M. Jansonius, Christopher J. Hammond, Jelle Vehof

**Affiliations:** Departments of * Medical Biochemistry; and; † Plastic and Reconstructive Surgery, Oslo University Hospital, Oslo, Norway;; ‡ Department of Ophthalmology, University of Groningen, University Medical Center Groningen, Groningen, the Netherlands;; § Department of Ophthalmology, Oslo University Hospital, Oslo, Norway;; ¶ Department of Epidemiology, University of Groningen, University Medical Center Groningen, Groningen, the Netherlands;; ‖ Department of Twin Research & Genetic Epidemiology, King's College London, St Thomas' Hospital, London, United Kingdom;; ** Department of Ophthalmology, King's College London, St Thomas' Hospital, London, United Kingdom;; †† Dutch Dry Eye Clinic, Velp, the Netherlands;; ‡‡ Department of Ophthalmology, Vestfold Hospital Trust, Tønsberg, Norway; and; §§ Departments of Ophthalmology and Epidemiology, University of Groningen, University Medical Center Groningen, Groningen, the Netherlands.

**Keywords:** dry eye, caffeine, coffee, tea, tear film, lifelines

## Abstract

The aim of this study was to determine the association between caffeine intake and dry eye disease (DED) in the large, population-based LifeLines cohort in the Netherlands.

**Methods:**

DED was cross-sectionally assessed in 85,302 participants (59% female participants) using the Women's Health Study dry eye questionnaire. Dietary caffeine was calculated from the intake of coffee, tea, cola, and energy drinks. Logistic regression was used to investigate the relationship between DED and caffeine, correcting for demographic variables, smoking status, alcohol intake, and 48 comorbidities of DED.

**Results:**

The mean (SD; range) age of participants was 50.7 years (12.4; 18–96), and 50,339 (59%) were female. The mean (SD) caffeine intake was 285 (182) mg/d. After correcting for demographics, body mass index, smoking status, and alcohol intake, higher caffeine intake was associated with a decreased risk of Women's Health Study-defined DED [odds ratio (OR) 0.971 per 100 mg/d, 95% CI, 0.956–0.986, *P* < 0.0005]. When additionally adjusting for medical comorbidities, no significant effect was observed (OR 0.985, 95% CI, 0.969–1.001, *P* = 0.06). Caffeine's effect on DED was similar in male and female participants and independent of sleep quality and stress at work. Decaffeinated coffee intake was significantly associated with an increased risk of DED, when adjusted for caffeinated coffee, demographics, alcohol intake, smoking status, and comorbidities (OR 1.046 per cup/d, 95% CI, 1.010–1.084, *P* = 0.01). None of the beverages were significantly associated with the risk of DED, when correcting for intake of the other caffeinated beverages, demographics, smoking status, alcohol intake, and all comorbidities.

**Conclusions:**

Dietary caffeine intake does not seem to be a risk factor for DED in the general population.

## Abstract 2

Supplemental Digital Content is Available in the Text.

Dry eye disease (DED) is a prevalent and multifactorial condition, affecting 5% to 50% of people.^1^ DED stems from a loss of ocular surface homeostasis, tear film instability, and hyperosmolarity.^2^ Dry eye symptoms, including irritation, dryness, and foreign body sensation, affect 1-in-4 patients seeking optometric care^3,4^ and are among the most common reasons for ophthalmological visits.^5^ There are substantial social and economic burdens associated with DED.^6^ In addition to pain and discomfort, people with DED have worse sleep quality,^7^ reduced quality of life,^8,9^ and impaired work productivity.^10^ Costs of DED in the United States are estimated to be more than 50 billion USD annually.^11^

Discovering modifiable risk factors is essential for effective treatment and prevention of DED. Known risks include computer use, systemic medications, and contact lens wear.^1^ Our group recently found alcohol intake to be tied to more dry eye symptoms in women^12^; however, the role of other dietary factors is still largely unknown. Caffeine (1,3,7-trimethylxanthine) is the most commonly ingested bioactive substance,^13^ but its role in DED development remains unclear, with inconclusive results in epidemiological^14–16^ and clinical studies.^17–19^ Determining caffeine's relationship with DED could provide guidance to patients and clinicians. In the US adults, coffee, tea, colas, and energy drinks account for 97% to 99% of dietary caffeine.^20^ Caffeine stimulates the central nervous system by antagonizing adenosine,^21,22^ which otherwise inhibits the neuronal activity and regulates the sleep and wake cycle.^23^ Adenosine receptors are present in the eye^24^ and may affect lacrimal gland secretion.^25,26^ However, caffeine's effects on the ocular environment are still largely unknown.

This study aimed to evaluate the relationship between dietary caffeine intake and DED in the large, population-based LifeLines cohort in the Netherlands. The large sample size allowed for accounting for demographic variables, smoking status, alcohol intake, and a wide range of comorbidities.^27^

## MATERIALS AND METHODS

### LifeLines Cohort and Participants

LifeLines is a multidisciplinary, prospective, population-based cohort study examining the health and health-related behaviors of 167,729 persons living in the north of the Netherlands. It uses a broad range of investigative procedures in assessing the biomedical, sociodemographic, behavioral, physical, and psychological factors which contribute to the health and disease of the general population, with a special focus on multimorbidity and complex genetics.^28^ Participants, almost exclusively of the European ancestry, were included by general practitioners or self-enrollment between 2006 and 2013 and will be followed for at least 30 years. The cohort is described in detail elsewhere.^29^ The study protocol was approved by the medical Ethics Committee of the University Medical Center Groningen and conducted in accordance with the Declaration of Helsinki, and all participants provided written informed consent.

**FIGURE 1.**
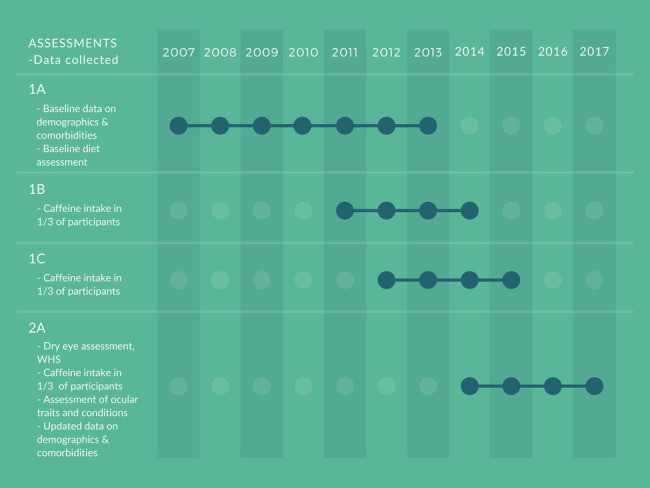
Timeline of the assessment of dry eye, caffeine intake, and comorbidities. Dry eye was assessed using the WHS questionnaire at 2A, whereas caffeine intake was assessed in one third of the participants at each of the timepoints 1B, 1C, and 2A.

The first general assessment in 2007 to 2013 (1A) was followed by 2 questionnaires after, on average, 1.5 years (1B) and 2.5 years (1C). A second general assessment occurred in 2014 to 2017 (2A). Dry eye was assessed at 2A, whereas caffeine intake and confounding factors were assessed either simultaneously (2A) or at earlier timepoints (1A–C). Figure 1 provides an overview of the timeline and what information was gathered from each assessment.

### Dry Eye Assessment

The Women's Health Study (WHS) dry eye questionnaire,^30^ the most used tool for assessing DED in population-based studies,^1^ was used. It has been validated against standardized clinical examinations and has sensitivity and specificity similar to a 16-item instrument.^30^ The questions are as follows: 1) “How often do your eyes feel dry (not wet enough)?” 2) “How often do your eyes feel irritated?” and 3) “Have you ever received a diagnosis of dry eye?” Questions 1 and 2 have possible answers such as “never,” “sometimes,” “often,” and “constantly.” Question 3 has possible answers such as “yes,” “no,” and “I don't know.”

The main outcome was WHS-defined DED, which is the presence of either a clinical diagnosis of DED or a ‟highly symptomatic dry eye” (see below).^30,31^ We further defined 3 secondary outcomes: 1) ‟clinical diagnosis of DED,” 2) ‟highly symptomatic dry eye,” and 3) ‟symptomatic dry eye,” as in past works.^12^ Participants who answered “yes” to having received a clinical diagnosis of dry eye were defined as having a ‟clinical diagnosis of DED.” Highly symptomatic dry eye was defined as having symptoms of both dryness and irritation at least “often,” whereas symptomatic dry eye included everyone with symptoms of dryness and irritation “sometimes” or either symptom at least “often.”

### Assessment of Caffeine Consumption

Dietary caffeine was assessed using flower-petal food frequency questionnaires developed by Wageningen University and Research.^32^ Combined, the questionnaires cover ≥96% of intake and ≥93% of interperson variability in nutrients.^32^ At baseline (1A), major food groups and total macronutrient intake were investigated. At each following assessment (1B, 1C, and 2A), one third of participants completed a questionnaire including detailed questions on dietary caffeine. These were completed, on median, 13 months (Interquartile range 0–27 mo) before DED assessment (2A).

Caffeine intake was calculated from the 4 major sources: coffee, tea, caffeinated cola, and energy drinks.^20,33^ Caffeine was computed from d/mo of consumption, units/d consumed, and caffeine/unit. The Netherlands Nutrition Centre's numbers for beverage caffeine content were used^34^ (see Supplemental Table 1, Supplemental Digital Content 1, http://links.lww.com/ICO/B358). Minor sources of caffeine, such as chocolate,^33^ and nondietary sources, including caffeine pills and adjuvant analgesics,^35^ were excluded. Imputation with the mean was used to account for missingness in tea (N = 1760), cola (N = 1054), and energy drink (N = 11,428) intake for all participants with complete data on coffee consumption.

### Assessment of Possible Confounding Factors

At baseline (1A), participants were asked: “Could you indicate which of the following disorders you have or have had?” Possible answers included a wide range of cardiovascular, chronic-pain, gastrointestinal, kidney and urinary, neurological, hematological, autoimmune, skin, and mental conditions. Any nonlisted disorders were reported using free text. At follow-ups, the occurrence of new conditions was investigated. A specific questionnaire about ocular traits and conditions was further administered concurrent with the DED assessment (2A). Dichotomous variables for the presence of a broad range of conditions were created, as described in greater detail elsewhere.^27^ Forty-eight comorbidities were associated with increased risk of WHS-defined DED.

### Statistics

The characteristics of the population were assessed with descriptive statistics. Multivariable logistic regression models were used to determine the relationship between the dichotomous DED outcomes and continuous caffeine intake (independent variable, base unit 100 mg/d). Model 1 included the dependent variable (DED outcomes), the independent variable continuous caffeine intake (in 100 mg/d), and covariables age and sex. Model 2 consisted of the DED outcomes and continuous caffeine intake, age, sex, education level (low, middle, or high), and net monthly household income (< 2000, 2000–3000, >3000 euros/mo), body mass index (BMI), self-reported alcohol intake in grams/d, and smoking status (never, current, or history of smoking). Model 3 included all variables in model 2 and the 48 medical comorbidities associated with WHS-defined DED.^27^

Because the prevalence and risk factors of DED are highly sex-specific,^12,36^ analyses were conducted both combined and sex-stratified. The interaction term (sex*caffeine intake) was included in regression models including all participants to assess the significance of any sex-specific relationship. Because sleep quality is tied to caffeine intake and DED risk, stratified analyses were conducted, using a Pittsburgh Sleep Quality Index scores ≥5.5 as a cut- off, in line with past works.^7^ Caffeine can be related to stress at work,^37^ which could trigger DED.^38^ Therefore, stratified analysis based on self-reported stress at work, assessed from the question “In the past year, to what extent did you experience difficulties and stress related to this aspect of your life?/At or with work,” was conducted. Finally, we assessed the intake of each beverage separately in a multivariate model.

Subsequently, the intake of caffeinated and decaffeinated coffee was analyzed in a separate multivariate model. This was performed because coffee and tea contain several other bioactive substances, including chlorogenic acid in coffee^39^ and catechins in tea.^40^ Multicollinearity between independent variables was checked. A *P*-value under 0.05 was regarded as statistically significant for analysis of the main outcome. However, because 3 secondary outcomes were included, Bonferroni correction was used, and a significance level of 0.05/3 (≈0.0167) was applied for the secondary outcomes. All analyses were conducted using SPSS software, version 25.0 (SPSS Inc).

## RESULTS

Eighty-five thousand three hundred two participants were included in this study. Table 1 summarizes the characteristics of the included participants. Nine percent of the included participants had WHS-defined DED. The mean caffeine intake was 285 mg/d. Coffee was the primary caffeine source, accounting for 92% and 89% of caffeine intake in men and women, respectively. Tea was the second-largest source, providing 6% of caffeine intake in men and 10% in women. Nearly all (98%) had a caffeine intake more than 0 mg/d, and 85% ingested at least 85 mg caffeine (equivalent to 1 cup of coffee) per day.

**TABLE 1.** Characteristics of the Study Population

	All (N = 85,302)	Men (N = 34,963)	Women (N = 50,339)
Age, yr, mean (SD)	50.7 (12.4)	51.6 (12.5)	50.1 (12.3)
Ethnicity—White, European, %	98.6%	98.8%	98.4%
Income			
<2000 Euro per mo	27.3%	21.6%	31.2%
2000–3000 Euro per mo	29.6%	33.1%	27.2%
>3000 Euro per mo	33.1%	37.6%	30.0%
Chose not to answer	10%	7.7%	11.6%
Smoker			
Current	15.6%	17.0%	14.6%
Former	33.3%	35.7%	31.2%
Never	51.1%	47.3%	54.2%
Dry eye			
WHS definition, %	9.0%	5.0%	11.9%
Highly symptomatic dry eye, %	1.9%	0.9%	2.6%
Clinical diagnosis	8.4%	4.6%	11.1%
Symptomatic dry eye, %	30.0%	22.4%	35.3%
Comorbidities*			
No. comorbidities, mean (SD)	2.9 (2.1)	2.3 (1.8)	3.2 (2.3)
Presence of ≥1 comorbidity	88.9%	85.0%	91.7%
Caffeine intake			
Caffeine intake, mg/d, mean (SD)	285 (182)	339 (190)	249 (167)
High caffeine intake (≥400 mg/d), %	24.7%	35.1%	17.5%
Coffee consumption			
Coffee intake, cups/d, mean (SD)	3.4 (2.2)	4.1 (2.2)	3.0 (2.0)
Intake of ≥1 cup/d of coffee	83.9%	90.8%	79.1%
Tea consumption (N = 82,170)			
Tea intake, cups/d, mean (SD)	2.1 (2.0)	1.4 (1.6)	2.5 (2.1)
Intake of ≥1 cup/d of tea	61.1%	46.0%	71.3%
Cola consumption (N = 79,957)			
Cola intake, glass/d, mean (SD)	0.3 (0.8)	0.4 (0.9)	0.3 (0.7)
Intake of ≥1 cup/d of colas	10.2%	13.8%	7.6%
Energy drink consumption (N = 73,938)			
Energy drink intake, glass/d, mean (SD)	0.01 (0.1)	0.02 (0.1)	0.01 (0.1)
Intake of ≥1 cup/d of energy drinks	0.2%	0.3%	0.2%

* Contact lens wear, hypertension (measured), macular degeneration, glaucoma/ocular hypertension, eye surgery (any), allergic conjunctivitis, Bell palsy, keratoconus, laser refractive surgery, irritable bowel syndrome, fibromyalgia, osteoarthritis, spinal disc herniation, repetitive strain injury, rheumatoid arthritis, systemic lupus erythematosus, Sjogren disease, atherosclerosis, cardiac arrhythmia, liver cirrhosis, chronic cystitis, urinary incontinence, spasticity, migraine, chronic fatigue syndrome, depression, burnout, autism, gastric ulcer, Crohn's disease, asthma, acne, psoriasis, eczema, rosacea, hay fever, allergy (any), anemia, diabetes mellitus, osteoporosis, thyroid disease (any), Graves disease, carpal tunnel syndrome, obstructive sleep apnea, lichen planus, sarcoidosis, chronic back pain, or sinusitis.

Table 2 lists the association between caffeine intake and all 4 phenotypes of dry eye. Increasing caffeine intake was associated with a reduced risk of the main outcome variable, WHS-defined DED, when correcting for age and sex only (model 1), as well as in model 2 including additional demographic variables, BMI, alcohol intake, and smoking status. However, after additional adjusting for comorbidities (model 3), no significant association between caffeine intake and DED was observed. Greater intake of caffeine was tied to fewer clinical diagnoses of DED in all the models. In models 1 and 2, symptomatic dry eye became less prevalent with higher caffeine consumption, whereas no relationship was observed for highly symptomatic dry eye. However, when adjusting for all comorbidities (model 3), no significant relationship was seen between caffeine and symptomatic dry eye, whereas increased caffeine intake was tied to more highly symptomatic dry eye.

**TABLE 2.** Relationship Between Caffeine Intake (Per 100 mg/d) and Dry Eye Phenotypes in the Total Population (N = 85,302)

Dry Eye Phenotypes	OR (95% CI), Model 1*	*P*	OR (95% CI), Model 2^†^	*P*	OR (95% CI), Model 3^‡^	*P*
Primary outcome						
WHS-defined DED	**0.966 (0.953–0.980)**	**<0.001**	**0.969 (0.954–0.984)**	**<0.001**	0.985 (0.969–1.001)	0.06
Secondary outcomes						
Highly symptomatic dry eye	1.018 (0.989–1.049)	0.22	1.017 (0.984–1.050)	0.32	**1.051 (1.017–1.086)**	**0.003**
Clinical diagnosis	**0.960 (0.946–0.974)**	**<0.001**	**0.965 (0.950–0.981)**	**<0.001**	**0.980 (0.964–0.996)**	**0.02**
Symptomatic dry eye	**0.985 (0.977–0.993)**	**0.001**	**0.988 (0.979–0.998)**	**0.02**	1.000 (0.990–1.010)	0.98

Bolded items indicate statistical significance (*P* < 0.05 for the primary outcome, WHS-defined DED, and *P* < 0.05/3 for secondary outcomes).

* Model 1: corrected for age and sex alone.

†Model 2: corrected for age, sex, body mass index, alcohol intake, smoking status, education level, and net monthly household income, full data available for 77,034 participants.

‡Model 3: corrected for age, sex, body mass index, alcohol intake, smoking status, education level, net monthly household income, and 48 comorbidities associated with dry eye; full data available for 75,032 participants.

Table 3 presents the results of the sex-stratified analyses. As in the main analysis, there was a lower risk of WHS-defined DED with greater caffeine intake in models 1 and 2, but no significant relationship in model 3 for both men and women. Overall, caffeine seemed to affect men and women similarly, and the interaction term (Caffeine intake*sex) was not significant for WHS-defined DED in any of the analyses.

**TABLE 3.** Relationship Between Caffeine Intake (per 100 mg/d) and Dry Eye Phenotypes, Adjusted for all Associated Comorbidities, and Stratified by Sex

Dry Eye Phenotypes	Men (N = 34,963)						Women (N = 50,339)					
	OR (95% CI), Model 1*	*P*	OR (95% CI), Model 2†	*P*	OR (95% CI), Model 3‡	*P*	OR (95% CI), Model 1*	*P*	OR (95% CI), Model 2†	*P*	OR (95% CI), Model 3‡	*P*
Primary outcome												
WHS-defined DED	**0.954 (0.930–0.980)**	**<0.001**	**0.965 (0.937–0.993)**	**0.02**	0.982 (0.954–1.012)	0.24	**0.967 (0.951–0.984)**	**<0.001**	**0.967 (0.950–0.985)**	**<0.001**	0.983 (0.964–1.002)	0.08
Secondary outcomes												
Highly symptomatic dry eye	1.004 (0.946–1.066)	0.89	1.007 (0.943–1.075)	0.85	1.025 (0.959–1.096)	0.47	1.017 (0.983–1.052)	0.32	1.013 (0.977–1.051)	0.48	**1.056 (1.016–1.097)**	**0.006**
Clinical diagnosis	**0.950 (0.924–0.976)**	**<0.001**	**0.961 (0.933–0.990)**	**0.01**	0.979 (0.950–1.010)	0.18	**0.960 (0.944–0.977)**	**<0.001**	**0.963 (0.945–0.982)**	**<0.001**	0.977 (0.958–0.997)	0.03
Symptomatic dry eye	**0.973 (0.960–0.986)**	**<0.001**	**0.978 (0.964–0.993)**	**0.004**	0.990 (0.975–1.005)	0.20	0.989 (0.978–1.000)	0.05	0.991 (0.979–1.003)	0.15	1.004 (0.991–1.018)	0.51

Bolded items indicate statistical significance (*P* < 0.05 for the primary outcome, WHS-defined DED, and *P* < 0.05/3 for secondary outcomes).

* Model 1: corrected for age and sex alone.

† Model 2: corrected for age, sex, body mass index, alcohol intake, smoking status, education level, and net monthly household income, full data available for 77,034 participants.

‡ Model 3: corrected for age, sex, body mass index, alcohol intake, smoking status, education level, net monthly household income, and 48 comorbidities associated with dry eye, full data available for 75,032 participants.

Table 4 presents the association between caffeine intake and WHS-defined DED, stratified by sleep quality and stress at work. The relationship between caffeine and DED seemed to be independent of both factors. Before adjusting for comorbidities (model 2), greater caffeine intake was associated with a reduced risk of DED across all strata. In model 3, only those experiencing stress at work showed a significant relationship between increased caffeine and reduced DED.

**TABLE 4.** Relationship Between Caffeine Intake (Per 100 mg/d) and WHS-Defined DED, Stratified by Sleep Quality and Stress at Work

Good Sleepers (PSQI <5.5) (N = 58,262, Mean Caffeine Intake: 290 mg/d)				Poor Sleepers (PSQI≥5.5) (N = 13,222, Mean Caffeine Intake: 268 mg/d)			
OR (95% CI), Model 2*	*P*	OR (95% CI), Model 3†	*P*	OR (95% CI), Model 2*	*P*	OR (95% CI), Model 3†	*P*
0.976 (0.957–0.994)	**0.01**	0.988 (0.969–1.008)	0.23	**0.967 (0.937–0.998)**	**0.04**	0.983 (0.951–1.016)	0.30

“Not” Stressed at Work (N = 50,104, Mean Caffeine Intake: 285 mg/d)				“Slightly” or “Very” Stressed at Work (N = 26,368, Mean Caffeine Intake: 288 mg/d)				
OR (95% CI), Model 2*	*P*	OR (95% CI), Model 3†	*P*	OR (95% CI), Model 2*	*P*	OR (95% CI), Model 3†	*P*
**0.975 (0.956–0.994)**	**0.01**	0.992 (0.972–1.012)	0.41	**0.952 (0.926–0.9878)**	**<0.001**	**0.966 (0.940–0.994)**	**0.02**

Bolded items indicate statistical significance (*P* < 0.05).

* Model 2: corrected for age, sex, body mass index, alcohol intake, smoking status, education level, and net monthly household income.

† Model 3: corrected for age, sex, body mass index, alcohol intake, smoking status, education level, net monthly household income, and 48 comorbidities associated with dry eye, N (good sleepers) = 57,547, N (poor sleepers) = 12,966, N (not stressed at work) = 49,163, N (stress at work) = 26,218.

PSQI, pittsburgh sleep quality index.

Not all sources of caffeine had a similar relationship with DED. Table 5 presents the association between WHS-defined DED and units/d of the 4 caffeine sources. Only increasing coffee consumption was associated with a reduced risk of DED in any of the models. Tea consumption was associated with a greater risk of DED in both models 1 and 2. In addition, decaffeinated coffee was associated with an increased risk of having DED in all 3 models, as given in Table 6.

**TABLE 5.** Relationship Between WHS-Defined DED and Units Per Day of the Sources of Caffeine, in a Multivariate Model Including All Sources

	All (N = 70,666)					
	OR (95% CI), Model 1*	*P*	OR (95% CI), Model 2†	*P*	OR (95% CI), Model 3‡	*P*
Coffee intake (cups/d)	**0.984 (0.970–0.998)**	**0.03**	0.987 (0.972–1.002)	0.09	1.000 (0.984–1.016)	0.98
Tea intake (cups/d)	**1.027 (1.013–1.042)**	**<0.001**	**1.021 (1.006–1.036)**	**0.006**	1.013 (0.998–1.029)	0.09
Cola intake (glass/d)	0.975 (0.928–1.024)	0.31	0.970 (0.919–1.023)	0.26	0.950 (0.898–1.004)	0.07
Energy drink intake (glass/d)	1.183 (0.950–1.471)	0.13	1.124 (0.875–1.443)	0.36	1.005 (0.758–1.333)	0.97

Bolded items indicate statistical significance (*P* < 0.05).

* Model 1: corrected for age and sex alone.

† Model 2: corrected for age, sex, body mass index, alcohol intake, smoking status, education level, and net monthly household income, full data available for 65,834 participants.

‡ Model 3: corrected for age, sex, BMI, alcohol intake, smoking status, education level, net monthly household income, and 48 comorbidities associated with dry eye; full data available for 64,924 participants.

**TABLE 6.** Relationship Between WHS-Defined DED and Cups/d of Caffeinated/Decaffeinated Coffee in Coffee Drinkers Only, in a Multivariate Model Including Both

	All (N = 56,332)					
	OR (95% CI), Model 1*	*P*	OR (95% CI), Model 2†	*P*	OR (95% CI), Model 3‡	*P*
Caffeinated coffee intake (cups/d)	**0.975 (0.961–0.990)**	**0.001**	**0.981 (0.965–‡0.997)**	**0.02**	0.995 (0.979–1.012)	0.58
Decaffeinated coffee intake (cups/d)	**1.034 (1.002–1.068)**	**0.04**	**1.042 (1.007–1.077)**	**0.02**	**1.046 (1.010–1.084)**	**0.01**

Bolded items indicate statistical significance (*P* < 0.05).

* Model 1: corrected for age and sex alone.

† Model 2: corrected for age, sex, body mass index, alcohol intake, smoking status, education level, and net monthly household income; full data available for 52,041 participants.

‡ Model 3: corrected for age, sex, body mass index, alcohol intake, smoking status, education level, net monthly household income, and 48 comorbidities associated with dry eye; full data available for 51,607 participants.

## DISCUSSION

In this large epidemiological study, greater dietary caffeine intake was not tied to an increased risk of WHS-defined DED. When correcting for relevant demographics, smoking status, alcohol intake, and BMI, higher caffeine intake was tied to a mildly reduced risk of DED. However, no relationship was observed when also adjusting for medical comorbidities. Caffeinated coffee was the only source of caffeine independently associated with decreased DED risk. Both tea and decaffeinated coffee were linked with increased risk of DED. The effects of caffeine were similar in male and female participants and independent of the participants' sleep quality and stress at work.

This is the first large epidemiological study focusing on the relationship between total caffeine intake and DED. In a smaller epidemiological study assessing only coffee consumption in 9752 Korean adults, Jeong et al^16^ found clinically diagnosed DED to be less prevalent in those drinking more coffee. However, this was not significant when correcting for age and sex.

Caffeine's role as a risk factor for dry eye symptoms has been assessed in general prevalence and risk factor studies, with differing results. Greater caffeine consumption (calculated from coffee, tea, cola, and hot chocolate) was associated with reduced age- and sex-adjusted prevalence of self-reported DED in the Beaver Dam Eye Study (N = 3722).^14^ However, the authors found no association with 5-year^41^ and 10-year incidence^15^ of DED in the same sample. No association between caffeinated beverage intake and dry eye symptoms was found in 2 small population-based studies.^42,43^ Higher coffee consumption was tied to a lower risk of having a DED diagnosis among symptomatic participants in Japan.^44^ However, coffee intake did not reduce the odds of having severe symptoms of DED in their subsequent study.^45^ Others have found that drinking more caffeinated beverages was associated with a decreased prevalence of DED.^46^ Better tear film break-up times and phenol red thread test scores were found in Australian women consuming more caffeinated beverages daily.^47^

Collectively, the past studies support the results of the current study. When correcting for all comorbidities, caffeine was related to an increased risk of highly symptomatic dry eye but a decreased risk of having a DED diagnosis. It is possible that caffeine affects dry eye symptoms separately from tear secretion and ocular surface parameters. Caffeine's role in pain modulation is complex.^48,49^ Caffeine inhibits adenosine receptors that modulate peripheral and central pain and has analgesic properties in moderate doses.^49,50^ However, at lower doses, caffeine blocks the analgesic effect of other compounds,^51^ and long-term effects are unclear.

In addition, caffeine may have direct ocular effects and seems to overall stimulate lacrimal gland secretion.^17,18^ In a placebo-controlled study assessing the effect of intake of pure caffeine, Schirmer I scores increased from baseline and compared with placebo.^17^ This was in contrast to an earlier uncontrolled study, which found instant coffee ingestion to decrease Schirmer I scores.^19^ In another placebo-controlled study, pure caffeine yielded a significantly greater increase in tear meniscus height than placebo.^18^ This effect was found to be significantly affected by single nucleotide polymorphisms in the genes for adenosine A2a receptor (*ADORA2A*) and cytochrome P450 1A2 (*CYP1A2*).^18^ CYP1A2 is the main enzyme in caffeine metabolization.^52,53^ The adenosine receptor family is found in several different regions of the eye,^24^ and ADORA2A is key in caffeine-induced wakefulness.^54^ Antagonism by caffeine could promote acetylcholine release^55,56^ leading to increased lacrimal gland secretion,^25,26,57^ promoting greater tear volumes.

Some have speculated that caffeine's diuretic properties may promote DED development through dehydration^58^ and thus advise reduced consumption. However, the habitual intake of caffeine does not have a significant diuretic effect.^59^ Caffeinated beverages have similar hydrating qualities as still water,^60^ and urine production and hydration status seem to be similar after ingestion of 1 L of either still water, instant coffee, tea, or caffeinated soda.^61^ Thus, it is unlikely that dehydration from caffeine would play a role in DED development.

Adjusting for medical comorbidities removed the significance of the inverse association between caffeine intake and WHS-defined DED. However, this could stem from overcorrection because caffeine and/or coffee can reduce the risk of several comorbidities,^27^ including blood pressure, cardiovascular disease, diabetes, and depression.^62–67^ Even with this possible overcorrection, caffeine was significantly related to a lower risk of a clinical diagnosis of DED. Thus, it is unlikely that caffeine is a risk factor for DED development in the general public. However, it should be noted that patients with a diagnosis of DED might reduce their intake or abstain from caffeine, affecting this relationship.

This study had several limitations. Because this is a cross-sectional assessment, the causality of the association cannot be determined. In addition, caffeine intake was assessed through a self-reported food frequency questionnaire. This method is naturally prone to recall bias, although bias is relatively low for habitually consumed beverages, such as coffee, tea, and alcohol.^68–70^ The absence of clinical tests for DED is another limitation. A possible weakness was that caffeine intake was not assessed at the same time point for all participants and that imputation by the mean was used to account for missing data in some categories. However, sensitivity analyses showed that these factors did not affect the results (data not shown).

An important weakness of this study is that the effects of caffeine could not be distilled because the assessed beverages contain several other bioactive compounds. Coffee is rich in terpenoids and trigonelline,^71,72^ and the main dietary source of chlorogenic acids,^73^ which are anti-inflammatory.^74^ However, increased intake of decaffeinated coffee was tied to a greater, not reduced, risk of DED, possibly indicating that the observed effect stemmed from caffeine rather than other substances in coffee.

Strengths of this study include the use of a validated questionnaire for the assessment of DED and the assessment of dietary caffeine from all major sources.^33^ Furthermore, the large sample size allowed for stratified analyses and multivariable models with many possible confounding factors. Moreover, this study was able to evaluate the impact of decaffeinated and caffeinated coffee separately, revealing nuances in the potential impact of caffeine versus other bioactive substances in coffee. Finally, because of the large sample size and rich dataset, it was possible to assess other possible confounders, such as sleep quality and stress at work, further clarifying the relationship.

Based on the results of this large, population-based, cross-sectional study, dietary caffeine does not seem to be a risk factor for DED. Despite a mildly increased risk of highly symptomatic dry eye after adjusting for many comorbidities, increasing caffeine intake was still found to slightly reduce the risk of having a DED diagnosis. No increased risk of having WHS-defined DED was found. Based on current evidence, discouraging caffeine intake in patients with DED on a general basis is not recommended.

## Supplementary Material
