# Supplementary material for: The Relationship Between Caffeine Intake and Dry Eye Disease
Source: Cornea. 2022 Jan 26;42(2):186–93. doi: 10.1097/ICO.0000000000002979 (PMC9797200; doi:10.1097/ICO.0000000000002979)
Supplement: Supplementary file 1 [file cornea-42-186-s001.docx]

**Supplementary Table 1: Overview of caffeine content per unit of beverage used.**

| **Coffee** |  |
| --- | --- |
| - Caffeinated Coffee | 85 mg/cup |
| - Decaffeinated Coffee | 3 mg/cup |
| **Tea** |  |
| - Black Tea | 30 mg/cup |
| - Green Tea | 30 mg/cup |
| - Other Tea | 0 mg/cup |
| **Cola** |  |
| - Caffeinated Cola | 18 mg/glass |
| - Decaffeinated Cola | 0 mg/glass |
| **Energy drinks** | 80 mg/can |
